# The various clinical spectra of juvenile xanthogranuloma: imaging for two case reports and review of the literature

**DOI:** 10.1186/s12887-019-1490-y

**Published:** 2019-04-24

**Authors:** Michaela Höck, Bernhard Zelger, Gisela Schweigmann, Barbara Brunner, Bettina Zelger, Gabriele Kropshofer, Ursula Kiechl-Kohlendorfer

**Affiliations:** 1 Department of Paediatrics II Neonatology, Medical University of Innsbruck, 6020 Innsbruck, Austria; 2 Department of Dermatology and Venerology, Medical University of Innsbruck, Innsbruck, Austria; 3 Department of Radiology, Medical University of Innsbruck, Innsbruck, Austria; 4 Department of Pathology, Medical University of Innsbruck, Innsbruck, Austria; 5 Department of Paediatrics I Oncology, Medical University of Innsbruck, Innsbruck, Austria

**Keywords:** Juvenile xanthogranuloma, Non-Langerhans cell histiocytosis, Blueberry muffin baby, Case report, Systemic, Histopathology

## Abstract

**Background:**

Juvenile xanthogranuloma (JXG) belongs to the heterogeneous group of non-Langerhans cell histiocytosis and is caused by an accumulation and proliferation of macrophages. In the majority of cases JXG is a disorder of early childhood presenting during the first 2 years of life. The typical presentation is a solitary reddish or yellowish skin papule or nodule with spontaneous regression and no need for treatment.

**Case presentation:**

Two infants with an atypical presentation of JXG, one with multiple blueberry muffin rash-like skin lesions and the other with severe multi-systemic involvement, are reported. Diagnosis was established by skin biopsy including histological work-up and immunostaining, where markers for macrophages (CD68 and CD163) exhibited significant reactivity.

**Conclusion:**

JXG is the most common of the non-Langerhans cell histiocytosis. The typical presentation is a solitary skin lesion. The purpose of this report is to familiarize paediatricians with an unusual variant of this entity in order to facilitate early diagnosis and raise awareness for possible visceral complications and associated medical conditions.

## Background

Juvenile xanthogranuloma (JXG) is a rare ‘histiocytic’ disorder and belongs to the broad group of non-Langerhans cell histiocytosis [1]. As noted in a report of this condition by Helwig and Hackney in 1954, Rudolf Virchow was the first to describe a child with cutaneous xanthomas in 1871 [2]. Other early reports of JXG were published in 1905 by Adamson [3] and in 1912 by McDonagh [4]. The real incidence is unknown, but it may be higher than is generally appreciated, because JXG is often underdiagnosed, in particular in people with dark skin. In the Kiel Paediatric Tumor Registry spanning 35 years JXG accounted for 129 (0.5%) out of 24.600 paediatric lesions. It is predominantly a disease of infancy or early childhood with a median age of onset between 5 months and 1 year [5], but congenital-type juvenile xanthogranuloma is also reported [6]. More males are affected than females, with a ratio of 1.4:1. JXG may affect all ethnicities, but few black patients with JXG have been reported [7]. Pathogenesis of JXG has not been uncovered, however it is most likely a reactive and not a neoplastic process. Kitchen et al. assumed a disordered macrophage response resulting from a nonspecific injury [8].

A triple association of juvenile xanthogranuloma, neurofibromatosis Type I (NF1) and juvenile myelomonocytic leukaemia (JMML) is often reported, but is the subject of frequent debate. In 2004 Burgdorf and Zelger analysed the literature and all available information pertaining to the association and found that patients with NF1 are, indeed, at an increased risk for developing JMML and JXG, but that the triple association of these findings (assuming the worst odds) is < 1% per year. However, regardless of the presence of JXG, children with NF1 are at a 200 to 500-fold greater risk for this hematologic malignancy. With regard to these rare events, lesions of JXG and NF1 may sometimes be clinically very similar and difficult to differentiate without histology. Moreover, lesions of JXGs and skin infiltrates of JMML may sometimes also be difficult to differentiate, clinically as well as histologically, all of which has significant influence on these statistical considerations [9]. There are also limited reports of the coexistence of JXG and cytomegalovirus infection [10].

Histopathology, clinical presentation and prognoses show great diversity. The presumed cell of origin of cutaneous JXG is a macrophage, derived in skin from the dermal dendrocyte, which represents a mixed dermal infiltrate of mononuclear cells, multinucleated giant cells and spindle cells [11]. Immunostaining is important in establishing the diagnosis: JXG stains positive for factor XIIIa, CD68, CD163, CD14 and fascin and is mostly negative for S100 protein and regularly negative for CD1a and anti-langerin (CD207), which are specific for Langerhans cells [12]. The typical clinical feature is a solitary, reddish or yellowish-tanned papule, plaque or nodule with a size of 0.5–2 cm, which generally appears on the head, neck, or trunk. Nevertheless, lesions can occur at any location in the body including lung, liver, spleen, lymph nodes, gastrointestinal tract, heart, kidney, bone marrow and central nervous system [13]. Also eye involvement is described [14]. For skin lesions, spontaneous regression within 1 to 5 years is the rule and treatment is rarely required [15]. JXG with systemic (extracutaneous) involvement is an uncommon disorder in which significant morbidity and occasional death may occur. Implications for the patient’s condition depend on the degree of visceral dysfunction from the benign mass. Therefore, therapy must be initiated when JXG interferes with vital organ functions. Various treatment strategies including chemotherapy (LCH-III protocol, a Langerhans cell disease-based regimen including corticosteroids and vinca alkaloids) [16], surgical resection [17] and radiation are reported.

To illustrate the various spectra of JXG we present two completely different cases, the way to reach a diagnosis, the clinical course, treatment and differential diagnoses of both cases.

## Case presentation

### Patient 1

**Fig. 1:**
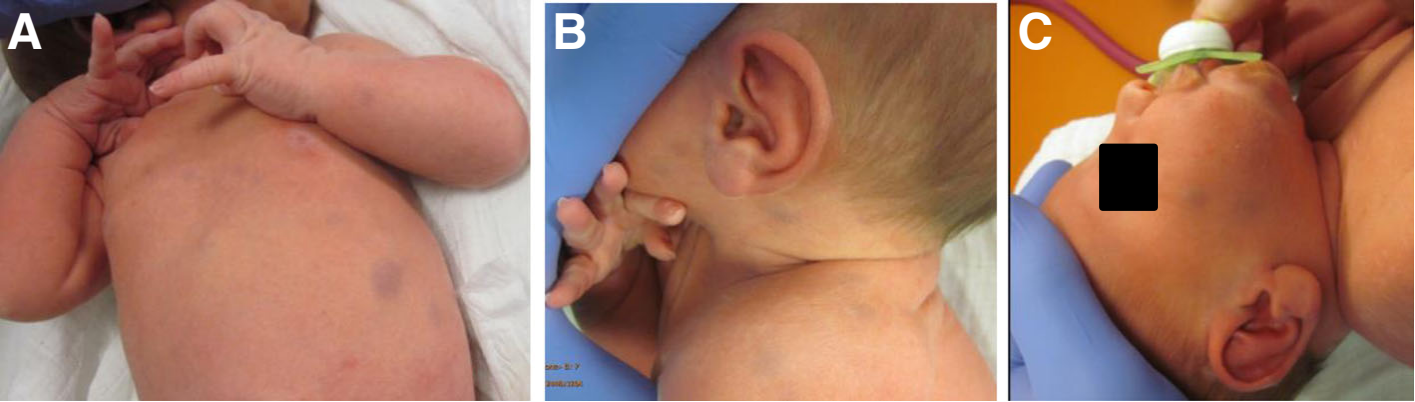
**a**, **b** and **c** Patient 1, a newborn boy with “blueberry muffin”-like skin rash

**Fig. 2:**
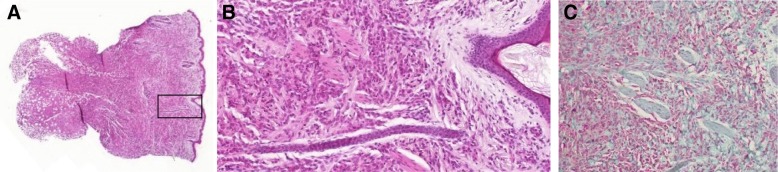
Histological appearance. Skin biopsy specimen from lesion on back. **a** (HE × 4) shows nodular to diffuse infiltrate of dermis and subcutis, **b** (HE × 100) monomorphous vacuolated macrophages without significant atypia or atypical mitoses, sparse presence of eosinophils and sparing of papillary and periadnexal dermis (Shapiro variant of xanthogranuloma), **c** CD163 immunohistochemistry with strong cytoplasmic reactivity (× 100)

**Fig. 3:**
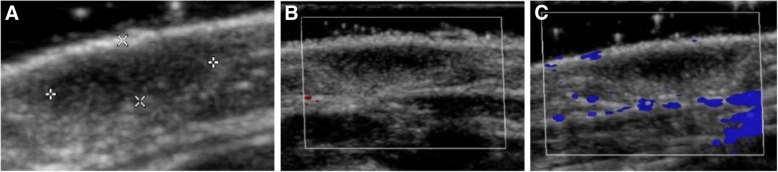
**a** Ultrasound of cutis/subcutis showed an ovaloid, hypoechoic change in the cutis, diameter 0.8, **b** and **c** Colour Doppler image shows no vascularity

A newborn boy, the second child (Fig. 1) of healthy, non-consanguineous, Caucasian parents was born in the 37 + 6th gestational week after an uncomplicated pregnancy. Birth weight was 3210 g (75th percentile), length 48 cm (20th percentile) and head circumference 35 cm (75th percentile). Postnatal adaptation was good with Apgar scores of 10/10/10 and an umbilical cord of pH 7.2. Clinical examination showed multiple magenta- to purple-coloured macules, papules and blueberry muffin-like lesions located on the trunk, face and extremities. Their size varied from 0.5 to 1 cm. Clinical examination was unremarkable, and especially there was no hepatosplenomegaly or lymphadenopathy. Routine laboratory studies including haematological and biochemical parameters were within the normal range. Due to the “blueberry-muffin” rash an extensive infectiological work-up, including TORCH screening, was undertaken with negative results. Excision biopsy of a lesion was performed and the diagnosis of congenital juvenile xanthogranuloma was established (Fig. 2). Imaging (Fig. 3) detected no systemic involvement. Therefore, a wait-and-see strategy was recommended. At the age of 10 months the patient was in complete remission and there is still no evidence of disease after 3 years.

### Patient 2

**Fig. 4:**
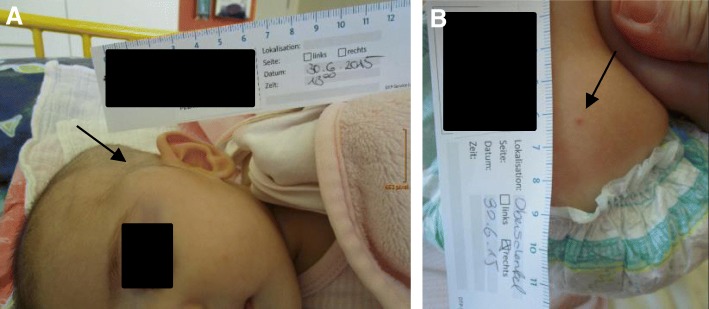
Patient 2, a 3-month-old-girl, **a** intraosseous soft tissue lesion in the left sphenoid bone, **b** cutaneous lesion at presentation

**Fig. 5:**
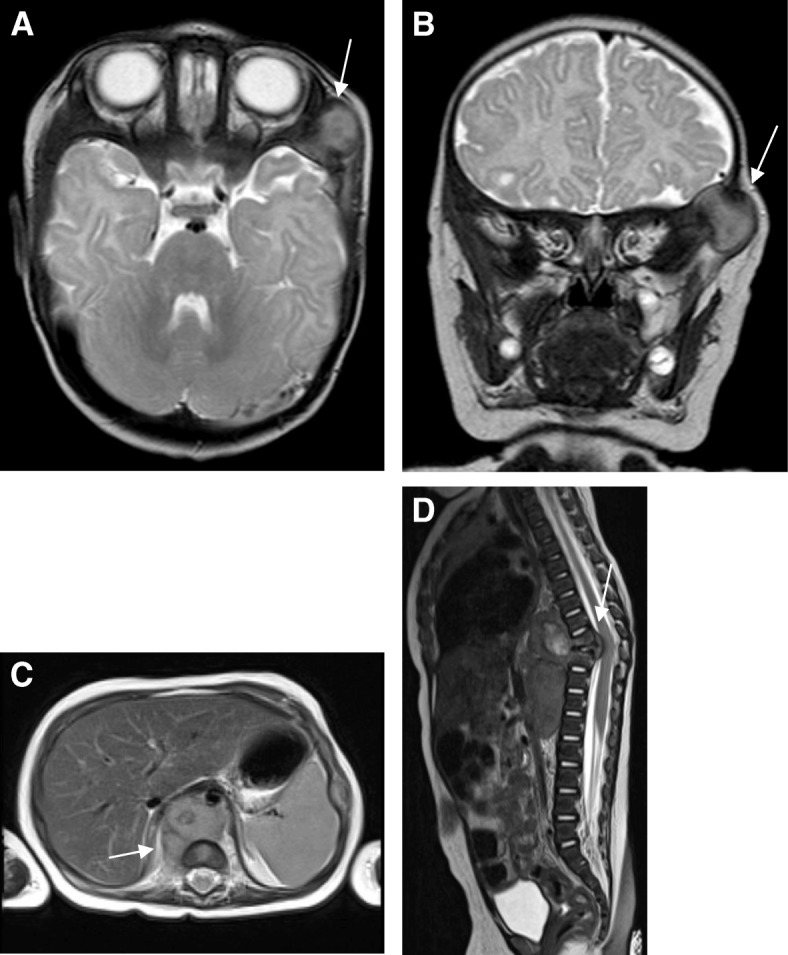
Magnetic resonance imaging. **a** head transversal and **b** head coronal T2 TSE: intraosseous soft tissue mass in the left sphenoid bone. **c** trunk axial T2 BLADE: prevertebral mass with elevation of diaphragm and thoracal and abdominal aorta. **d** spine sagittal T2 TSE: vertebra plana Th9, adjacent Th8 and Th10 wedge-shaped, thoracal gibbus, compromise of spinal cord by intraspinal part of Th9

**Fig. 6:**
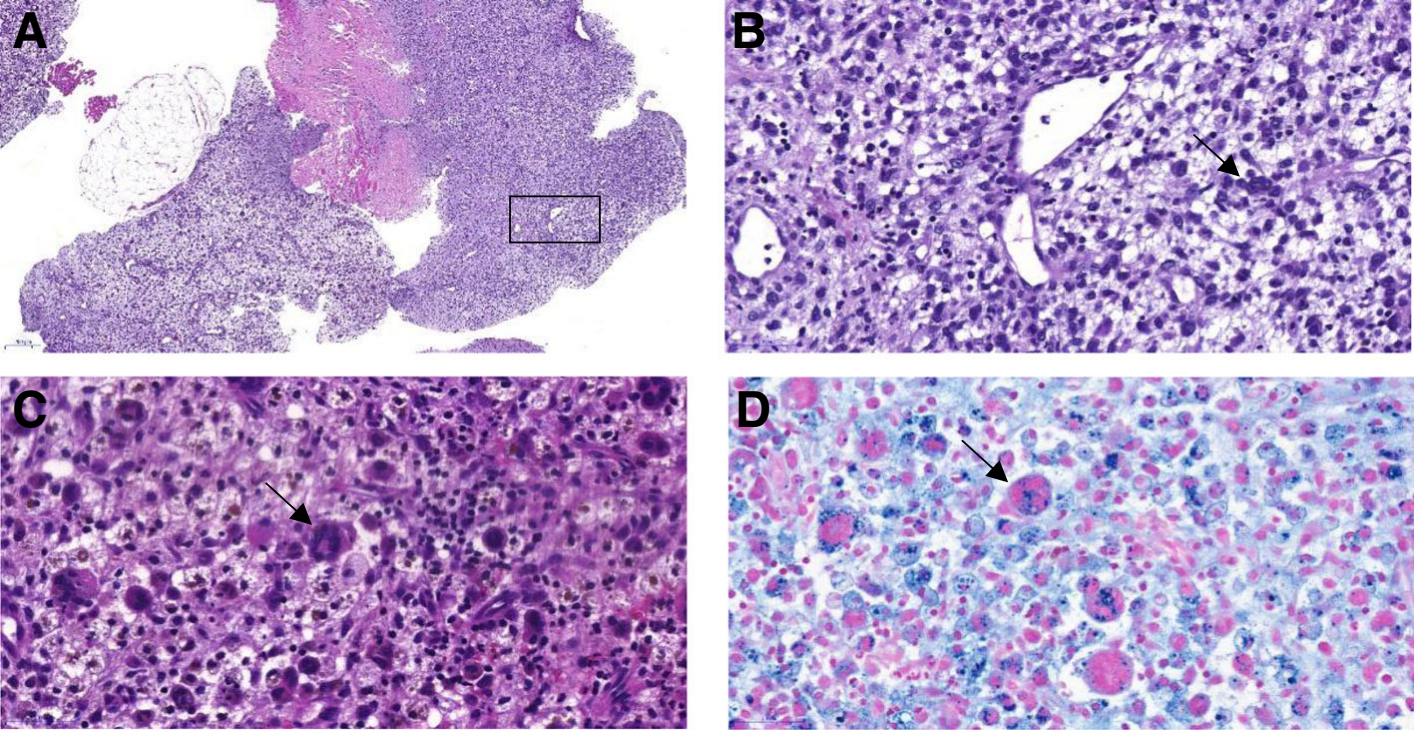
Histological appearance. Skin biopsy specimen from the sphenoidal bone. **a** (HE × 140) Besides fatty and striated muscle tissue nodular to diffuse infiltrate of vacuolated and oncocytic (plasmocytoid) as well as mostly xanthomatized (foamy) mononuclear and multinucleate macrophages, which in the first place gives the lesion a more dense eosinophilic appearance, and in the second place a faint colour. **b** (HE × 200) High power from area indicated in **a** nicely outlines xanthomatized cells. **c** (HE × 200) shows oncocytic/plasmocytoid mononuclear cells with dense amphophilic ground glass cytoplasm, occasional eosinophils as well as some Touton and ground glass giant cells, some of the latter with moderate emperipolesis indicated by arrow as well as presence of a prominent brown pigment, which in **d** (Prussian stain × 200) reveals siderophages, a phenomenon of xanthogranulomas in the literature known as xanthosiderohistiocytosis

The second child (Fig. 4) of a 29-year-old woman was spontaneously born at 39 + 4 weeks of gestation after an unremarkable pregnancy. Birth weight was 3510 g (50th percentile), length 55 cm (75th percentile) and head circumference 33.5 cm (20th percentile). Apgar scores were 9/10/10. At the age of 3 months the girl was seen by a general pediatrician and consecutively referred to our hospital because of a recently developed mass on the left temple. The subcutaneous swelling was about 2 cm in diameter, non-moveable, not reddish or overheated and not painful. Furthermore, the mother reported recurrent fever spikes up to 38.5 °C without signs of inflammation for about 4 weeks. Defecation and drinking habits were adequate, vomiting was denied. However, a weight loss of 200 g within 3 weeks was obvious. In addition to a pale skin color and three pinhead-large livid subcutaneous lesions located on the trunk and the lower extremities, there was a left-sided rib hump situated at the level of Th6 to Th10; a secondary finding was oral candidiasis. Laboratory values on admission showed: hemoglobin 85 g/l, hematocrit 0.24 L/l, thrombocytes 380 G/l, lactate dehydrogenase 308 U/l, alpha-1-fetoprotein 225.6 ng/ml, beta-human chorionic gonadotropin < 1 mU/ml, c-reactive protein 10.13 mg/dl, interleukin-6 45.8 pg/ml and procalcitonin 0.31 ng/ml. To define the extent of disease, whole-body magnetic resonance imaging (MRI) (Fig. 5) was performed. An intraosseous soft tissue lesion in the left sphenoid bone (diameter 18 × 20 mm), a big paravertebral thoracic tumor conglomerate (diameter 85 × 59 mm), multiple papules to nodules in the liver (7 mm), in both kidneys (6 mm) and lungs (3 × 4.3 mm) and in the pancreatic head (3.5 mm), as well as cutaneous (5 mm) and intraosseous lesions were found. A vertebra plana of Th9, together with infiltration of the adjacent Th8 and Th10, resulting in a kinking of the spinal column compromising the spinal canal and obliteration of nerve roots by soft tissue tumor mass was seen. Due to the lesion in the skull and the vertebra plana, Langerhans cell histiocytosis was one of the primary differential diagnoses. But the histology of one cutaneous lesion of the trunk did not confirm this diagnosis. Rapid deterioration with paraplegia prompted us to administer immunosuppressive treatment immediately. Based on the presumed diagnosis of a neoplasia of the Ewing / PNET group the patient was initially treated according to the Euro-Ewing protocol. After the third biopsy and histological examination two independent pathology centers confirmed the diagnosis of xanthosiderohistiocytosis, which is not well-defined and is regarded as a morphologic variant of xanthoma disseminatum – a type that most often occurs in adult patients with monoclonal gammopathy (Fig. 6). In keeping with the established diagnosis, Langerhans cell histiocytosis-based chemotherapy treatment was administered. Following the arm for the high-risk group, the chemotherapy agents included prednisone, vinblastine, 6-mercaptopurine and methotrexate. With this therapy the primary tumor mass decreased. Clinical and radiologic examinations at the age of 3 years show partial remission after 1 year maintenance chemotherapy with puri-nethol and methotrexate.

## Discussion and conclusions

### General

Cutaneous juvenile xanthogranuloma is a common ‘histiocytic’ disorder, but a detailed review of the literature reveals only a small number of cases of systemic juvenile xanthogranulomatosis in the neonatal period [18] and less than 15 cases of spinal JXG [19]. Although cutaneous JXG is generally regarded as a self-limited condition, systemic JXG may be associated with significant morbidity and occasional deaths so that aggressive medical care is necessary [20]. To illustrate this point, we report on two affected children, both born within 1 year in Austria, who were confirmed to have JXG.

The originality of our observation is the clinically atypical and completely different presentation of this rare disease by the multi-lesional and multisystemic nature of its pathology. Moreover, it illustrates the difficulty of classifying this disorder, because the clinical and radiological presentation is nonspecific. Therefore, correlation with histopathology is mandatory and the gold standard for diagnosis of JXG.

### Clinical spectrum

In the first patient we describe cutaneous JXG, which follows a benign course and gradual regression of the lesion without treatment. The diagnosis was established quickly, although the skin lesions were not typical of JXG. The typical presentation is a solitary erythematous or yellowish, well-circumscribed skin papule on the head, neck or trunk. Our patient presented with blueberry-muffin spots. Excision biopsy of the lesions was performed and established the JXG diagnosis. The absence of the typical yellowish colour was due to the lack of xanthomatization because of lesion immaturity. Thus, this case together with four more case reports in the literature [21–24] indicates that the diagnosis of JXG should be included in the differential diagnosis of clinical presentation of a blueberry muffin baby.

With the second patient we report on one of the few documented cases (fewer than 45) of congenital systemic JXG [25], presenting with a reduced general condition, a mass on the temple, fever, weight loss, and discrete skin involvement. Because of typical lesions in MRI (lesion in the skull and vertebra plana) and difficulties obtaining a usable biopsy for adequate histopathological analysis, the diagnosis of systemic JXG was delayed for several weeks. Despite the fact that fatal cases of systemic JXG - particularly central nervous system and hepatic involvement - have been reported only rarely [26–29], prompt diagnosis and treatment are essential.

### Imaging

In accordance with other reports [30, 31] diagnostic work-up with ultrasound showed a well-defined, homogeneous, hypoechoic lesion without demonstrable blood flow in the dermis (Patient 1) or viscera (Patient 2) in both patients [30, 31].

Magnetic resonance imaging (1.5 T) demonstrated the broad extension of the disease. In the literature, enhancement is described as a reliable feature of JXG lesions [32]. The typical imaging ranges from iso- to hyperintense on T1 and iso- to hypointense on T2 [31, 33]. MRI findings in our Patient 2 showed the big thoracic tumor conglomerate on T1 and on T2 slightly hyperintense to muscle, furthermore multiple nodular lesions in the liver, hyperintense in TIRM and T2 and hypointense in T1-weighted sequences. MRI imaging is nonspecific and variable. However, it is the first option for localizing the lesion.

### Cytogenetics

The molecular cytogenetic findings in Patient 2 with systemic JXG showed 9p-(ptercen), 9p-(p21.3p21.1) and 9q rearrangements (9q33.3qter) positive, which could be a possible chromothripsis region involved in cancer and congenital diseases. The MYCN oncogene presented no indication for an amplification (2p/MYCN-negative). To date little is known about the genetic profile of juvenile xanthogranuloma. However, previous studies have reported that systemic JXG showed multiple genomic alterations, while solitary JXG usually has normal genomic profiles [34].

### Histopathologic features

Because of its typical clinical appearance, diagnosis of JXG is established clinically in most cases. However, its heterogeneous appearance may cause misdiagnosis. To confirm the clinical findings, skin biopsy for histology and immunostaining is essential. However, even this does not always provide a clear result, because more than 100 different subtypes of histiocytosis with a wide range of histological and immunohistochemical presentation have been described.

Classic histology of JXG shows a dense, sheet-like, noncapsulated, well demarcated cell infiltration in the dermis and the upper portion of the subcutaneous fat, while the epidermis and adnexal skin structures are spared. Cellular infiltrate includes five main cell types (vacuolated, xanthomatized, spindle-shaped, scalloped and oncocytic) in variable proportions (from monomorphous to mixed variants) with different types of giant cells (nonspecific, foreign body, Touton and “ground-glass”). Appearance mostly depends on the age of the lesion: while early lesions show a monomorphic infiltrate of lipid-free macrophages that can occupy most of the dermis, mature lesions contain abundant vacuolated, foamy macrophages and Touton-type multinucleated giant cells, particularly in the superficial dermis. Immunohistochemically, JXG lesions typically stain positive with macrophage markers including CD68, CD163, KiM1P, anti-FXIIIa, vimentin and anti-CD4 and usually are negative for S-100 protein and regularly negative for CD1a and CD207 (anti-langerin), which is specific for Langerhans cells [35].

In Patient 1 the lesion showed a diffuse infiltration of epithelioid cells, sparing the papillary dermis and periadnexal connective tissue. There were monomorphic vacuolated cells without cellular atypia or increased or atypical mitoses. The immunohistochemical findings (Fig. 2c) were negative for mast cell and Langerhans cell markers: S-100 protein, CD1a, CD207 (anti-langerin), toluidine blue histochemistry, c-kit (CD117). The markers for macrophages CD68 and CD163 exhibited significant reactivity.

In Patient 2 the diagnosis was much more difficult and required three biopsies for histological and immunohistochemical work-up - including a referral report - to get the correct diagnosis. The first biopsy, a skin punch, showed eosinophils with strong mitotic activity. Immunohistochemistry showed S-100 protein and CD99 positivity, while CD1a stained negative, typical for a neoplasia of the Ewing/PNET group. The second skin biopsy from the soft tissue lesion on the infant’s left temple was sent to a reference centre and showed sheets of foamy macrophages admixed with mononuclear cells and numerous multinucleated giant cells. There were admixed lymphocytes and neutrophils, and a very prominent stromal haemosiderin deposition. So-called xanthosiderohistiocytosis was regarded as a morphologic variant of xanthoma disseminatum. Small areas consisted of the more monomorphic mononuclear cells similar to those seen in the initial skin biopsy. There was no atypia or pleomorphism and mitoses were scarce. Immunostaining showed strong and diffuse positivity for CD163, while S-100 protein was negative. It was labelled as an unclassified benign xanthogranulomatous lesion. However, the appearances did not match well with that of a conventional juvenile xanthogranulomatous lesion, so we performed another – computed tomography – assisted - biopsy of the mass in the posterior mediastinum showing cellular infiltrates of foamy macrophages with prominent nucleoli and eosinophilic granulocytes. Immunohistochemical work-up demonstrated a homogeneous and intensive CD68 and CD163 positivity, while NSE and CD99 showed nonspecific reaction patterns. CD207 (anti-langerin) and CD1a as well as HMB-45 remained negative. S-100 protein showed isolated dendritic background cells; otherwise it remained mostly negative, except for a non-specific reaction in the macrophages. Thus, definitive diagnosis was xanthogranuloma or xanthogranulomatous reaction.

ALK immunoreactivity was observed in a novel type of systemic histiocytic proliferative disorder that may suggest a storage disorder and should be a possible marker for systemic involvement with xanthogranulomas [36]. We performed ALK immunostaining in our cases, which, however, was negative in both patients, so that we could not confirm the previous study [36] suggesting that ALK might be a marker for systemic involvement.

### Differential diagnoses

In Patient 1 the main symptom was the blueberry muffin-type rash, which is a potentially life-threatening condition with severe sequelae requiring extensive and prompt diagnostic work-up. Differential diagnoses can be divided into several broad categories: the first category includes haematological and non-haematological malignancies. Especially the differential diagnosis between JXG, in particular the Shapiro variant which is seen in this case, and cutaneous manifestations of JMML can be tricky and difficult to differentiate. The isolated myelosarcoma of skin in childhood is a rare manifestation of acute myeloid leukaemia preceding bone marrow involvement by weeks to months. Case reports in the literature describing the clinical presentation as blueberry muffin spots or symptoms of infection and anaemia are rare [37]. Histologically, most cases are classified as monoblastic or myelomonocytic leukaemia with atypical mitoses. Immunohistochemically, CD43 and lysozyme stain a large proportion of neoplastic cells, with MPO and CD117 being the most sensitive of markers for myeloid differentiation, while monocytic precursors consistently strongly express CD68 and CD163 [38]. Due to the small number of cases available for isolated myelosarcoma in children, prognostic statements are difficult. Spontaneous remission of congenital myelosarcoma is reported; however, the majority of cases progressed to AML within months. Taking into account the course of the disease in older patients, one could speculate that the prognosis is rather unfavourable. In synopsis of all findings, the benign clinical course of Patient 1 (at the age of 10 months the patient was in complete remission and after 3 years there is still no evidence of disease), the unremarkable laboratory findings (normal blood counts), the imaging (well-defined, homogeneous, hypoechoic lesion without vascularity), the histological (sparing of papillary dermis and periadnexal connective tissue as seen in our case, missing presence and number of (atypical) mitoses, low proliferation index with Ki-67) and immunohistochemical findings (positive for macrophage markers CD68 and CD163) the JXG diagnosis seems confirmed and valid. The second category includes congenital infections. However, TORCH work-up was negative in our patient. Finally, the third group includes extramedullary haematopoiesis in severe fetal and neonatal anaemia of any cause, but there was no evidence of a haemolytic disease like AB0 or Rh incompatibility or hereditary spherocytosis.

In Patient 2, histological and immunohistochemical findings were a little deceptive. JXG is mostly immunohistochemically negative for S-100 protein. However, case reports of S-100 protein-positive JXG were already reported in 1998 [39], complemented by a longitudinal observation study in 2009 [40], which demonstrates that S-100 protein reactivity cannot be reliably used as definitive marker for differentiating JXG from other histiocytoses, such as Rosai-Dorfman disease (RDD) or indeterminate cell histiocytosis. The latter also shows reactivity, with additional markers of Langerhans cells, namely CD1a and CD207 (anti-langerin), being absent in our cases. Both these entities frequently show the presence of eosinophils, which in our case were initially very prominent, in due course only very subtly present. Emperipolesis is a condition that can be observed in many physiological and pathological conditions, where hematopoietic cells in living and intact state are seen in the cytoplasm of the host cell without damage. Usually, JXG shows no emperipolesis. Yet, a high degree of emperipolesis in JXG, simulating Rosai-Dorfman disease, has been reported in individual series [41]. Macrophages in RDD are frequently foamy and can be multinucleated, so that they are difficult to differentiate from JXG. RDD derives from sinus histiocytic macrophages that are positive for S-100 protein, fascin, CD68, CD14, CD163 and HLA-DR and negative for CD1a and CD207. In our case another peculiarity of JXG may be helpful for delineation from RDD, namely iron deposition in siderophages. This phenomenon is well known for the reaction pattern of xanthogranuloma, then entitled xanthosiderohistiocytosis, but has to the best of our knowledge (so far) not been described in RDD.

## Conclusions

Juvenile xanthogranuloma belongs to the heterogeneous group of non-Langerhans cell histiocytoses and generally tends to have a good prognosis. However, the development of systemic disease can be detrimental if not diagnosed in a timely manner.

This report highlights the wide variety of clinical presentations: the first patient with an unusual skin manifestation, the second with visceral (lung, liver, pancreas, kidneys), skeletal (spine) and skin involvement and extension into soft tissue.

To make an early diagnosis and prompt adequate therapy it is pivotal, that all pediatricians be aware of this rare disease, because they are often the first to see these patients.

## Abbreviations

HE: Hematoxylin eosin
JMML: Juvenile myelomonocytic leukemia
JXG: Juvenile xanthogranuloma
LCH: Langerhans cell histiocytosis
MRI: Magnetic resonance imaging
NF1: Neurofibromatosis Type 1
NLD: Non-Langerhans cell disorder
PNET: Primitive neuroecodermal tumor
RDD: Rosai-Dorfman disease
TORCH: Acronym for toxoplasmosis, other (parvovirus B19, varicella zoster virus, listeriosis), rubella, cytomegalovirus, chlamydia, coxsackievirus, herpes simplex virus, hepatitis B/C virus, human immunodeficiency virus
